# Efficacy and outcome of molecular targeted therapies in elderly patients with hepatocellular carcinoma: Relative dose intensity associated with overall survival

**DOI:** 10.1002/cam4.6783

**Published:** 2023-12-07

**Authors:** Kyoko Oura, Asahiro Morishita, Kei Takuma, Mai Nakahara, Tomoko Tadokoro, Koji Fujita, Shima Mimura, Joji Tani, Masafumi Ono, Takashi Himoto, Tsutomu Masaki

**Affiliations:** ^1^ Department of Gastroenterology and Neurology, Faculty of Medicine Kagawa University Kagawa Japan; ^2^ Department of Medical Technology Kagawa Prefectural University of Health Sciences Kagawa Japan

**Keywords:** hepatocellular carcinoma, lenvatinib, molecular targeted agent, sorafenib, tyrosine kinase inhibitor

## Abstract

**Aim:**

Indications of drug therapies to elderly patients with hepatocellular carcinoma (HCC) should be carefully determined. The current study assessed the safety and efficacy of molecular targeted agents (MTAs) in the elderly patients with HCC, and identified factors associated with prognosis in a real‐world clinical setting.

**Methods:**

In a retrospective observational study, clinical data of patients with unresectable HCC treated with sorafenib or lenvatinib as first‐line treatment at our hospital between 2011 and 2022, were investigated. Clinical parameters, therapeutic effects, adverse events (AEs), and prognosis were evaluated separately for the non‐elderly (<75 years old) and elderly patients (≥75 years old).

**Results:**

Overall, 111 patients were enrolled, including 59 non‐elderly and 52 elderly patients. Compared to the non‐elderly patients, the elderly patients had significantly lower skeletal muscle mass and a significantly lower percentage of patients in poor general condition with performance status 2 or higher, but there were no differences in parameters related to liver function or nutritional status. There were no significant differences in the incidence of severe AEs and therapeutic effects between the groups. No significant difference in progression‐free survival was observed in the elderly and non‐elderly patients; however, overall survival (OS) for sorafenib treatment was shorter in the elderly patients than in the non‐elderly patients. Elderly patients consumed lower doses of both the drugs, and relative dose intensity (RDI) 4 weeks after treatment (4W‐RDI) was associated with OS. Further, OS in the elderly patients was significantly longer in the subgroup with high 4W‐RDI as compared to that in the subgroup with low 4W‐RDI.

**Conclusions:**

MTAs can be safely administered to elderly patients with HCC. Furthermore, 4W‐RDI is associated with longer OS. Maintaining RDI in the early phase is crucial in predicting the success of treatment with MTAs, especially in the elderly patients.

## 1 INTRODUCTION

Hepatocellular carcinoma (HCC) is the most common histologic type, accounting for more than 90% of primarily liver cancer cases.^1^ Recent epidemiological studies have demonstrated that the occurrence of HCC is increasing worldwide, not only in Europe and the United States, but also in West Asia and Africa with high hepatitis virus infection rates.^1^ Further, HCC ranked sixth in incidence and third in mortality among all cancer types in 2021.^2^, ^3^ The development and progression of HCC is known to be relevant to persistent hepatitis virus infection, autoimmune liver disease, alcoholic liver disease, nonalcoholic steatohepatitis, aflatoxin exposure, and multiple other risk factors.^1^, ^4^ In addition to curative treatments including liver resection and radiofrequency ablation, there have been remarkable advances in the treatment for intermediate‐ and advanced‐stage HCC, including transcatheter arterial chemoembolization (TACE) and molecular target agents (MTAs). However, the 5‐year survival rate for patients with HCC is still reported be poor, ranging from 15% to 38%,^5^, ^6^, ^7^ and is affected by a high recurrence rate and resistance to chemotherapy. HCC that has progressed with vascular invasion and/or extrahepatic metastasis is classified as stage C in the Barcelona clinic liver cancer (BCLC) staging system,^8^ and the median expected survival time for these patients is reported to be approximately 7–9 months.^9^ The systemic therapy with MTAs is indicated for advanced HCC, and several MTAs, such as sorafenib, regorafenib, lenvatinib, ramucirumab, and cabozantinib have shown favorable therapeutic effects and are used worldwide in the clinical setting.^10^ In addition, immunotherapy for advanced HCC is a promising strategy because immune checkpoint molecules including programmed death 1, programmed death‐ligand 1, and cytotoxic T‐lymphocyte antigen 4 are one of the common mechanisms of HCC immune escape in the microenvironment of HCC.^11^ To date, clinical studies have been conducted on the combination of immune checkpoint inhibitors (ICIs) with various agents. Among them, the combination of MTA and ICI is the latest therapy that plays a central role in treating advanced HCC.

Aging is a significant risk factor for hepatocarcinogenesis, and recent reports have indicated that the age at first diagnosis of patients with HCC is increasing in developed countries, including Japan, due to the global increase in the elderly population. In our previous study, the percentage of patients with HCC who were older than 75 years at diagnosis increased from 29.8% between 2003 and 2007 to 38.7% between 2013 and 2017.^12^ Elderly patients with HCC often have physical problems such as malnutrition and reduced skeletal muscle mass, and tend to be in poor general condition. Additionally, they experience complications from respiratory and cardiovascular diseases, or other malignancies, which require careful attention when determining the indications for and treating them. Furthermore, elderly patients have altered pharmacokinetics and pharmacodynamics due to polypharmacy,^13^ and several clinical trials for HCC treatment tend to include a low percentage of elderly patients. Although several MTAs are currently being prescribed to elderly patients in clinical practice, the small proportion of elderly patients with HCC in clinical trials of MTAs indicates that their therapeutic effects may not be universally replicated.

This study aimed to estimate the suitability of the elderly patients with HCC for systemic therapy, and to evaluate the efficacy of sorafenib and lenvatinib, the commonly used MTAs for HCC treatment. We also analyzed relative dose intensity (RDI), adverse events (AEs), clinical outcomes, and related clinical parameters to estimate their prognosis in real‐world clinical settings.

## 2 METHODS

### 2.1 Patients and study design

In this retrospective observational study, patients with unresectable HCC who were classified as stage B or C by the BCLC staging system, and started on sorafenib or lenvatinib as the initial MTA therapy from 2011 to 2022 at Kagawa University Hospital (Kagawa, Japan) were eligible for inclusion. Furthermore, patients treated with atezolizumab (Atezo) + bevacizumab (Bev) combination therapy were excluded since enough observation period could not be ensured. Patients who started other MTAs that are not recommended as first‐line agents were also excluded. These patients were classified into two categories according to age at the start of treatment: non‐elderly (<75 years) and elderly (≥75 years). Patient characteristics, therapeutic effects, drug dosage, AEs, and prognosis were compared between the two groups.

### 2.2 Evaluation of clinical parameters

HCC was comprehensively diagnosed based on tumor markers, contrast‐enhanced computed tomography (CT), magnetic resonance imaging, and the histological findings of a previous surgery. In the absence of typical HCC findings, needle biopsy was performed for definitive diagnosis. Other primary and metastatic liver cancers, including cholangiocarcinoma, were excluded. Patients' general condition was evaluated by assessing body mass index (BMI) and Eastern Cooperative Oncology Group Performance Status (PS).^14^ To assess skeletal muscle, the area of psoas muscle at the L3 level of the lumbar spine on CT images was measured using SYNAPSE VINCENT (FUJIFILM, Tokyo, Japan), and the area was divided by the square of the height to calculate psoas muscle index (PMI). According to the Japan Society of Hepatology guidelines for sarcopenia in liver disease, the cut‐off values were 6.36 cm^2^/m^2^ and 3.40 cm^2^/m^2^ for males and females, respectively.^15^, ^16^ Modified albumin‐bilirubin (mALBI) grade was used to assess liver function.^17^ Controlling the nutritional status (CONUT) score was calculated from serum albumin levels, serum cholesterol levels, and peripheral blood lymphocyte count to assess nutritional status.^18^ The Tumor‐Node‐Metastasis (TNM) classification based on the criteria of the Liver Cancer Study Group of Japan was used to determine the clinical stage based on tumor factors including number of tumor, tumor size, and vascular invasion, presence of lymph node metastasis, and presence of distant metastasis.^19^

### 2.3 Treatment protocol and assessment of AEs

The dose taken orally at the start of treatment in all patients was sorafenib 400 mg twice daily, or lenvatinib 12 mg (≥60 kg body weight) or 8 mg (<60 kg body weight) once daily; those who started at reduced doses were excluded. AEs were assessed by the Common Terminology Criteria for Adverse Events v5.0,^20^ and administration was discontinued when serious AEs or clinical tumor progression occurred. According to the pharmaceutical company dosing guidelines, the dose reduction or suspended until symptoms improved to Grade 1 or 2 when serious or unacceptable treatment‐related AE of Grade 3 or higher occurred. Furthermore, a stepwise dose adjustment of 400 mg once daily and 400 mg every other day was acceptable according to the manufacturer's guidelines for dose reduction due to AEs with sorafenib. For AEs with lenvatinib, a dose reduction to 8 mg/day, 4 mg/day, or 4 mg every other day was allowed after dose interruption. In addition, as a measure of treatment intensity, the RDI was calculated as the percentage of the actual dose of MTA relative to a predetermined standard dose at 4 and 8 weeks after the start of medication.

### 2.4 Evaluation of therapeutic effects

Contrast‐enhanced CT was performed at 2, 4, and 6 months of treatment. According to the modified Response Evaluation Criteria in Solid Tumors (mRESIST), the therapeutic effect was classified as complete response (CR), partial response (PR), stable disease (SD), and progressive disease (PD).^21^ The best therapeutic response during the observation period was adopted in analysis. Two or more radiologists were involved in the diagnosis of each case, and the diagnostic report prepared by an image‐reading physician was checked again by another diagnostic radiologist. The decision regarding the diagnosis of HCC, the selection of the most suitable treatment method, and the evaluation of therapeutic effects were discussed by hepatologists, oncologists, surgeons, and radiologists at our hospital in accordance with Japanese practice guidelines for HCC.^22^, ^23^

### 2.5 Statistical analysis

For statistical analysis, frequencies were compared using Fisher's exact tests or chi‐squared tests. For continuous variables, medians were calculated and differences were compared using Mann–Whitney *U*‐tests between two groups. Logistic regression analysis was performed on factors related to maintaining RDI. For prognosis, the Kaplan–Meier method was used to calculate the survival rate in September 2023, and the log‐rank test was used to examine significance. Furthermore, multivariate analyses were performed using the Cox proportional hazards model for factors associated with progression free survival (PFS) or overall survival (OS), and the Wald test was used to examine the significance of each factor. For these statistical analyses, JMP software (JMP Pro 14.2.0, Cary, North Carolina, USA) and GraphPad Prism (Prism 8.4.3, San Diego, California, USA) were used. Significance was set at *p* < 0.05.

### 2.6 Ethical approval

The protocol in this study was appropriately approved by the research ethical committee of Kagawa University Faculty of Medicine (ethics approval 2019‐238). Opt‐out consent form for this study is available on the Kagawa University website. Furthermore, this study was conducted in compliance with the Ethical Guidelines for Medical and Biological Research Involving Human Subjects, revised in 2022 by the Japanese Ministry of Health, and the Helsinki Declaration of 1975, as revised in 2008.

## 3 RESULTS

### 3.1 Patient characteristics

Overall, 111 patients were enrolled according to the protocol, including 59 non‐elderly and 52 elderly patients. Patient characteristics are presented in Table 1. The median age of the patients was 67 (43–74) and 80 (75–93) years in the non‐elderly and elderly groups, respectively. The median PMI between 5.05 (0.68–8.25) cm^2^/m^2^ and 4.92 (2.20–6.65) cm^2^/m^2^ in the non‐elderly and elderly groups. Moreover, 6.8% and 38.4% of non‐elderly and elderly patients, respectively, showed PS2 or higher, with a significantly higher proportion of elderly patients with HCC in poor general conditions. Regarding treatment, patients who received sorafenib and lenvatinib as first‐line regimen were 69.5% and 30.5% in non‐elderly group and 55.8% and 44.2% in the elderly group, respectively, with no significant difference. No differences were observed between the non‐elderly and elderly groups in parameters, including blood chemistry tests, tumor markers, mALBI grade, and CONUT score. There were no significant differences between the both groups in terms of tumor number of tumors, tumor size, major vascular invasion, extrahepatic metastasis, or clinical stage as classified by these parameters.

**TABLE 1:** Patient characteristics.

	Non‐elderly group	Elderly group	
Characteristic	*n* = 59	*n* = 52	*p*‐Value
Median age, years	67 (43–74)	80 (75–93)	< 0.01
Sex: Male, *n* (%)	43 (72.9)	43 (82.7)	N.S.
Etiology			
HBV/HCV/NBNC, *n* (%)	11/23/25 (18.6/39.0/42.4)	1/23/28 (1.9/44.2/53.8)	< 0.05
Median BMI, kg/m^2^	24.22 (16.85–36.64)	23.56 (17.99–33.70)	N.S.
Median PMI, cm^2^/m^2^	5.05 (0.68–8.25)	4.92 (2.20–6.65)	< 0.05
Performance status			
0/1/2/3, *n* (%)	46/9/4/0 (78.0/15.3/6.8/0.0)	9/23/19/1 (17.3/44.2/36.5/1.9)	< 0.01
First‐line regimen			
Sorafenib/Lenvatinib, *n* (%)	41/18 (69.5/30.5)	29/23 (55.8/44.2)	N.S.
Past history of TACE, *n* (%)	37 (62.7)	42 (80.8)	N.S.
Baseline bilirubin, mg/dL	0.8 (0.3–2.4)	0.7 (0.3–3.0)	N.S.
Baseline albumin, mg/dL	3.7 (2.0–4.7)	3.7 (2.3–4.9)	N.S.
Baseline α‐fetoprotein, ng/mL	42 (2–1,142,137)	46.5 (1–852,884)	N.S.
Baseline des‐γ‐carboxy prothrombin, mAU/mL	613 (13–1,625,624)	800 (13–605,642)	N.S.
mALBI grade			
1/2a/2b/3, *n* (%)	17/18/21/3 (28.8/30.5/35.6/5.1)	18/13/21/0 (34.6/25.0/40.4/0.0)	N.S.
CONUT score			
0–1/2–4/5–8/9–12, *n* (%)	13/30/15/1 (22.0/50.8/25.4/1.7)	7/25/19/1 (13.5/48.1/36.5/1.9)	N.S.
Maximum tumor size			
5 cm or less/greater than 5 cm, *n* (%)	37/22 (62.7/37.3)	33/19 (63.5/36.5)	N.S.
No. of tumors			
3 or less/4 or more, *n* (%)	16/43 (27.1/72.9)	14/38 (26.9/73.1)	N.S.
Major vascular invasion, *n* (%)	20 (33.9)	9 (17.3)	N.S.
Extrahepatic metastasis, *n* (%)	26 (44.1)	16 (30.8)	N.S.
TNM staging, LCSGJ 6th edition			
II/III/IVa/IVb, *n* (%)	2/21/12/24 (3.4/35.6/20.3/40.7)	4/25/8/15 (7.7/48.1/15.4/28.8)	N.S.

Abbreviations: BMI, body mass index; CONUT, controlling nutritional status; HBV, hepatitis B virus; HCV, hepatitis C virus; LCSGJ, Liver Cancer Study Group of Japan; mALBI, modified albumin‐bilirubin; N.S., not significant; NBNC, non‐hepatitis HBV and non‐HCV; PMI, psoas muscle index; TACE, transcatheter arterial chemoembolization; TNM, tumor‐node‐metastasis.

### 3.2 Therapeutic effects

The best therapeutic effects of each drug, according to mRESIST, are presented in Table 2. In the non‐elderly group, the objective response rate (ORR) (CR + PR) for sorafenib and lenvatinib treatments was 31.7% and 44.4%, respectively. In the elderly group, the ORR for sorafenib and lenvatinib was 13.8% and 30.4%, respectively, with both drugs demonstrating inferior response rates compared to non‐elderly patients, but not significant. Both the drugs demonstrated therapeutic responses comparable to previous clinical studies.^24^, ^25^, ^26^ Furthermore, lenvatinib demonstrated superior tumor reduction compared to sorafenib.

**TABLE 2:** Therapeutic effects evaluated by modified Response Evaluation Criteria in Solid Tumors.

	Non‐elderly group	Elderly group	*p*‐Value
Sorafenib	*n* = 41	*n* = 29	
ORR, *n* (%)	13 (31.7)	4 (13.8)	N.S.
CR, *n* (%)	4 (9.8)	3 (10.3)	N.S.
PR, *n* (%)	9 (22.0)	1 (3.4)	< 0.01
SD, *n* (%)	7 (17.1)	6 (20.7)	N.S.
PD, *n* (%)	21 (51.2)	19 (65.5)	N.S.
Lenvatinib	*n* = 18	*n* = 23	
ORR, *n* (%)	8 (44.4)	7 (30.4)	N.S.
CR, *n* (%)	2 (11.1)	1 (4.3)	N.S.
PR, *n* (%)	6 (33.3)	6 (26.1)	N.S.
SD, *n* (%)	4 (22.2)	9 (39.1)	N.S.
PD, *n* (%)	6 (33.3)	7 (30.4)	N.S.

Abbreviations: CR, complete response; N.S., not significant; ORR, objective response rate; PD, progressive disease; PR, partial response; SD, stable disease.

### 3.3 Drug dosage and AEs

Table 3 shows the tolerability of systemic therapy, including the dosage of MTAs and the occurrence of AEs. When treatment was initiated with sorafenib as the first‐line therapy, approximately half of both non‐elderly and elderly patients required dose reduction within 4 weeks. For sorafenib treatment, the median RDI in the first 4 weeks (4W‐RDI) in the non‐elderly and elderly groups was 50.0% and 44.6%, respectively. In addition, the median RDI at 8 weeks (8W‐RDI) was 31.3% and 24.6%, respectively, with significantly lower 4W‐RDI and 8W‐RDI in the elderly group throughout the study period. Furthermore, the occurrence of AEs in all grade was 92.7% and 86.2% in the non‐elderly and elderly groups with no significant difference, and no significant difference was observed in the occurrence of severe AEs between both groups. The most common AEs associated with sorafenib were hand‐foot syndrome, anorexia, liver failure, hypertension, and elevated liver enzyme levels, with a higher occurrence of anorexia in the elderly group, causing sorafenib dose reduction.

**TABLE 3:** Drug dosage and adverse events.

	Non‐elderly group	Elderly group	*p*‐Value
Sorafenib	*n* = 41	*n* = 29	
Drug administration			
Median RDI for 4 weeks, %	50.0 (16.1–100.0)	44.6 (6.3–58.9)	< 0.05
Median RDI for 8 weeks, %	31.3 (8.5–100.0)	24.6 (3.1–67.0)	< 0.05
AEs			
Any grade, *n* (%)	38 (92.7)	25 (86.2)	N.S.
Grade 3, *n* (%)	18 (43.9)	15 (51.7)	N.S.
Grade 4, *n* (%)	3 (7.3)	0 (0.0)	N.S.
Grade 5, *n* (%)	0 (0.0)	0 (0.0)	N.S.
Major AEs			
Hand‐foot syndrome, *n* (%)	10 (24.4)	5 (17.2)	N.S.
Decrease in appetite, *n* (%)	8 (19.5)	14 (48.3)	<0.05.
Liver failure, *n* (%)	7 (17.1)	5 (17.2)	N.S.
Hypertension, *n* (%)	6 (14.6)	2 (6.9)	N.S.
Elevated aspartate transferase, *n* (%)	3 (7.3)	4 (13.8)	N.S.
Lenvatinib	*n* = 18	*n* = 23	
Drug administration			
Median RDI for 4 weeks, %	100.0 (17.9–100.0)	71.4 (3.6–100.0)	N.S.
Median RDI for 8 weeks, %	74.9 (8.9–100.0)	50.0 (1.8–100.0)	< 0.05
AEs			
Any grade, *n* (%)	16 (88.9)	20 (86.9)	N.S.
Grade 3, *n* (%)	8 (44.4)	9 (39.1)	N.S.
Grade 4, *n* (%)	1 (2.4)	1 (4.3)	N.S.
Grade 5, *n* (%)	0 (0.0)	1 (4.3)	N.S.
Major AEs			
Hand‐foot syndrome, *n* (%)	6 (33.3)	4 (17.4)	N.S.
Platelet count decreased, *n* (%)	6 (33.3)	3 (13.0)	N.S.
Decrease in appetite, *n* (%)	4 (22.2)	14 (60.9)	<0.05
Liver failure, *n* (%)	3 (16.7)	2 (8.7)	N.S.
Hypertension, *n* (%)	4 (22.2)	7 (30.4)	N.S.

Abbreviations: AEs, adverse events; N.S., not significant; RDI, relative dose intensity.

Comparatively, fewer patients prescribed lenvatinib as first‐line agent required discontinuation or dose reduction of lenvatinib in the first 4 weeks compared to patients prescribed sorafenib. The median 4W‐RDI for lenvatinib was 100.0% and 71.4% in the non‐elderly and elderly groups, respectively, which was not significantly different. However, the median 8W‐RDI of lenvatinib administration was significantly different in non‐elderly and elderly groups at 74.9% and 50.0%, respectively. The incidence of AEs in all grades with lenvatinib was as high as that with sorafenib (88.9% and 86.9% in non‐elderly and elderly patients, respectively); however, the difference was not significant. In addition, the occurrence of severe AEs did not differ between the non‐elderly and elderly groups. The major types of AEs with lenvatinib were like those with sorafenib, with a higher occurrence of anorexia in the elderly group, causing lenvatinib dose reduction.

In the receiver operating characteristics analysis to detect PD with the best therapeutic effect, the optimal cutoff value of 4W‐RDI for sorafenib treatment was 39.3%. Sensitivity and specificity were 0.70 and 0.57, respectively. Furthermore, the optimal cutoff value of 4W‐RDI for lenvatinib treatment was 53.6%, with a sensitivity of 0.62 and specificity of 0.79. Using these approximate cutoffs, high RDI groups were defined as the patients with 4W‐RDI ≥40% for sorafenib and 4W‐RDI ≥50% for lenvatinib.

Logistic regression analysis showed that sex, age, past TACE, PS, mALBI grade, and PMI were not associated with high 4W‐RDI, but CONUT score 0–4 was the only factor significantly associated (Table S1). The association between 4W‐RDI and PS was analyzed by subgroups, divided into high RDI and low RDI in the elderly group. PS 0/1/2/3 was 4 (11.8%)/16 (47.1%)/13 (38.2%)/1 (2.9%) in the high 4W‐RDI subgroup (*n* = 34), while 5 (27.8%)/7 (38.9%)/6 (33.3%)/0 (0.0%) in the low 4W‐RDI subgroup (*n* = 18). There was no significant difference between these frequencies.

### 3.4 Frist‐line treatment discontinuation and second‐line treatment

**FIGURE 1:**
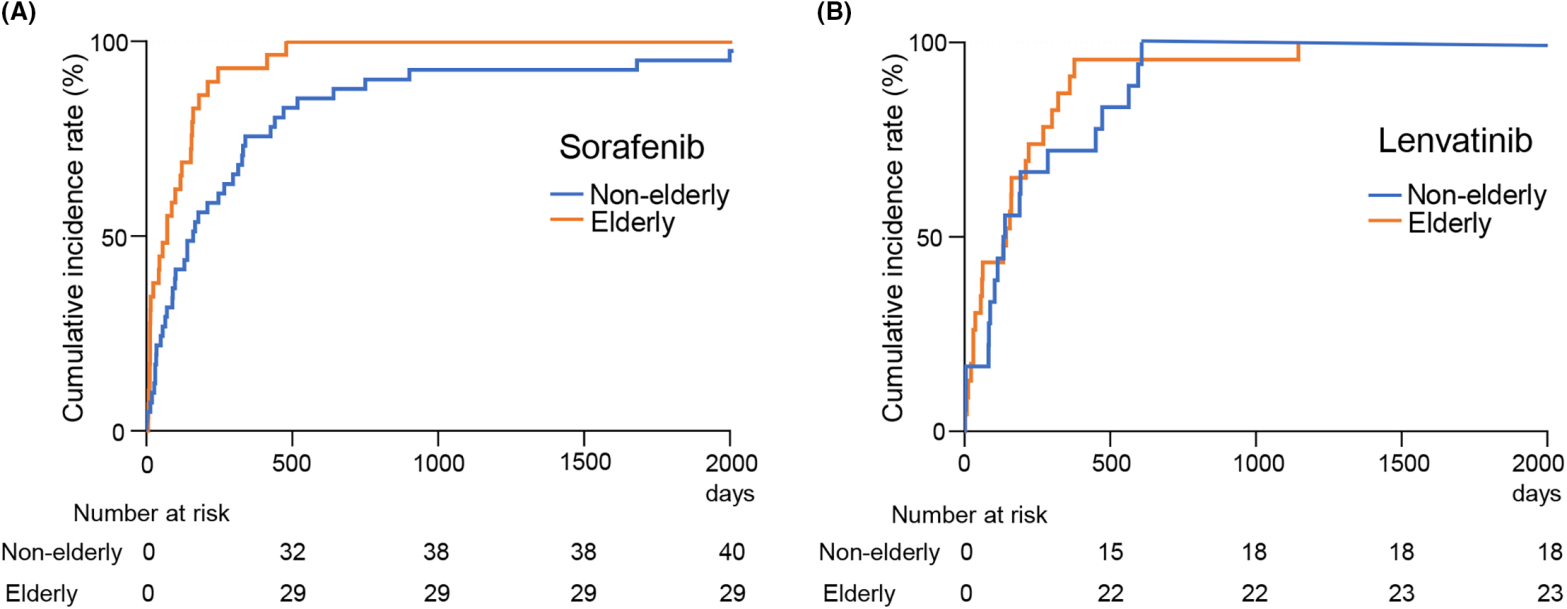
The cumulative incidence of first‐line treatment discontinuation. (A) For sorafenib treatment, the median duration of discontinuation was 161 and 72 days in the non‐elderly and elderly groups, respectively (**p < 0.01). (B) For lenvatinib treatment, the median duration of discontinuation was 136.5 and 143 days in the non‐elderly and elderly groups, respectively (not significant).

The cumulative incidence of discontinuation of first‐line treatment is shown in Figure 1. For sorafenib treatment, the median duration to discontinue the medication was 161 days in the non‐elderly and 72 days in the elderly group, a significant difference between the two groups (Figure 1A). The most common reason for discontinuation of sorafenib treatment was the occurrence of AEs in 48.3% of elderly patients. Further, 41.4% of elderly patients discontinued sorafenib administration due to PD. In contrast, the median duration to discontinue lenvatinib treatment was 136.5 and 143 days in the non‐elderly and elderly groups, respectively, with no significant difference (Figure 1B). In the elderly group, PD was responsible for lenvatinib discontinuation in 52.2%, and rest were due to AEs.

Next, second‐line treatment after discontinuation of first‐line treatment is presented in Table 4. The percentage of patients on sorafenib treatment who could progress to second‐line therapy was 36.6% and 31.0% in the non‐elderly and elderly groups, respectively; however, there were no significant differences in the regimens, including regorafenib, lenvatinib, or Atezo + Bev combination. On the contrary, the proportion of patients who could progress to second‐line therapy among those who started lenvatinib treatment was 44.4% and 52.2% in the non‐elderly and elderly groups, respectively. In addition, no significant differences were observed between the two groups with respect to the regimens that included sorafenib, ramucirumab, or an Atezo + Bev combination.

**TABLE 4:** The second‐line treatment.

	Non‐elderly group	Elderly group	*p*‐Value
Sorafenib	*n* = 41	*n* = 29	
Second‐line treatment, *n* (%)			
Yes	15 (36.6)	9 (31.0)	N.S.
No	26 (63.4)	20 (69.0)	
Regimen, *n* (%)			
Regorafenib	9 (22.0)	4 (13.8)	N.S.
Lenvatinib	4 (9.8)	3 (10.3)	
Atezo + Bev	2 (4.9)	2 (6.9)	
Lenvatinib	*n* = 18	*n* = 23	
Second‐line treatment, *n* (%)			
Yes	8 (44.4)	12 (52.2)	N.S.
No	10 (55.6)	11 (47.8)	
Regimen, *n* (%)			
Sorafenib	3 (16.7)	4 (17.4)	N.S.
Ramucirumab	2 (11.1)	2 (8.7)	
Atezo + Bev	3 (16.7)	6 (26.1)	

Abbreviations: Atezo, atezolizumab; Bev, bevacizumab; N.S., not significant.

### 3.5 Prognosis

**FIGURE 2:**
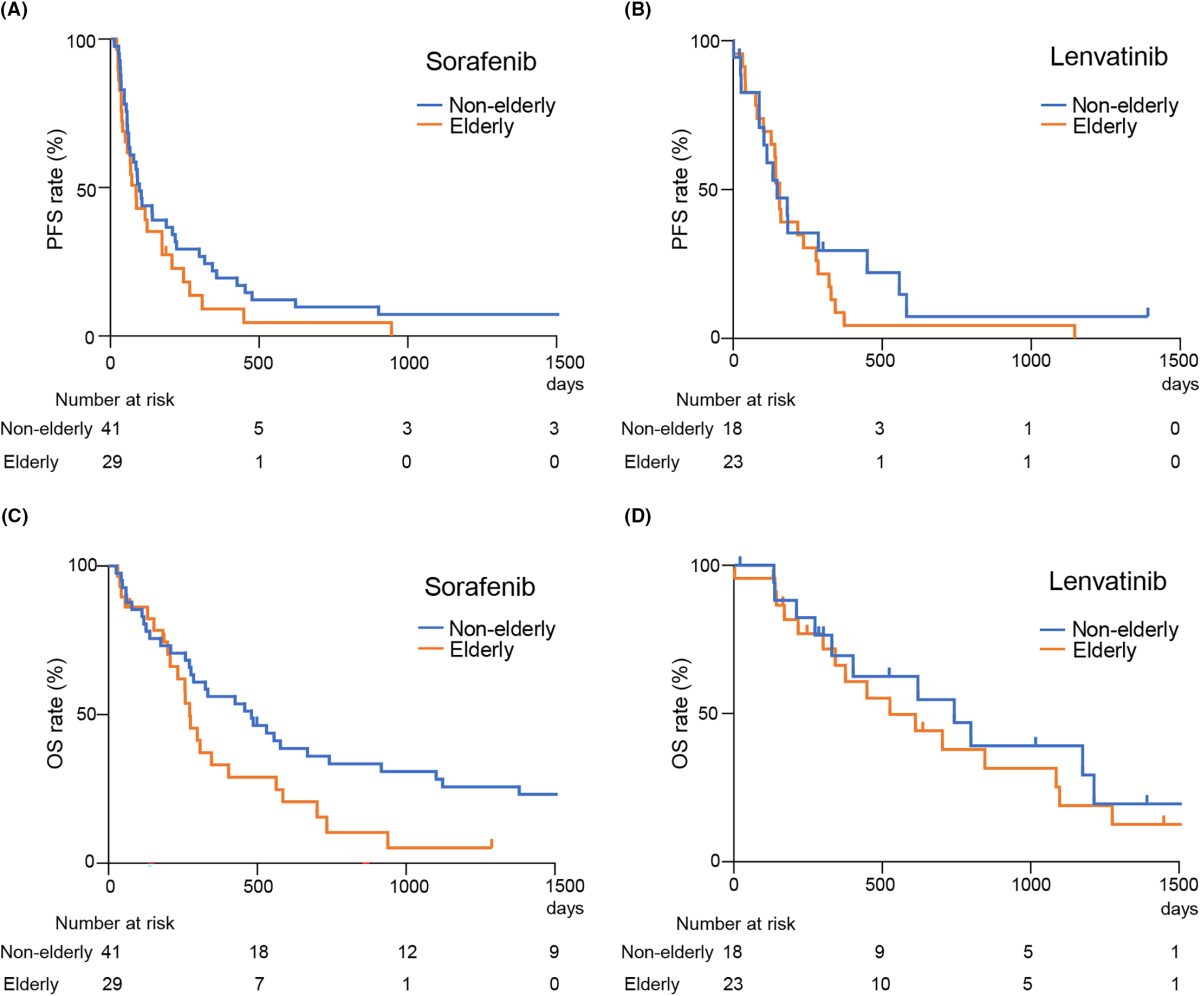
Progression‐free survival (PFS) and overall survival (OS) compared with non‐elderly and elderly patients. (A) The median PFS for sorafenib treatment was 98 and 86 days in the non‐elderly and elderly patients, respectively (not significant). (B) The median PFS for lenvatinib treatment was 148 and 156 days in the non‐elderly and elderly patients, respectively (not significant). (C) The median OS for sorafenib treatment was 480 and 273 days in the non‐elderly and elderly patients, respectively (*p < 0.05). (D) The median OS for lenvatinib treatment was 743 and 526 days in the non‐elderly and elderly patients, respectively (not significant).

PFS and OS are important outcomes for patients with unrespectable HCC treated with MTAs. The median PFS in patients who initiated first‐line treatment with sorafenib was 98 and 86 days in the non‐elderly and elderly groups, respectively, with no significant differences (Figure 2A). The median PFS for patients treated with lenvatinib was 148 and 156 days in the non‐elderly and elderly groups, respectively, with no significant differences (Figure 2B). Furthermore, the median OS in patients initiating first‐line treatment with sorafenib was 480 and 273 days in the non‐elderly and elderly groups, respectively, with the elderly having a significantly worse prognosis than non‐elderly patients (Figure 2C). In contrast, the median OS for patients initiating first‐line treatment with lenvatinib was 743 and 526 days in the non‐elderly and elderly groups, respectively, with a slight trend toward shorter OS in the elderly patients. However, the difference was not significant (Figure 2D). Notably, these results indicate that OS in elderly patients tends to be longer with lenvatinib as the first‐line agent in comparison to sorafenib.

**FIGURE 3:**
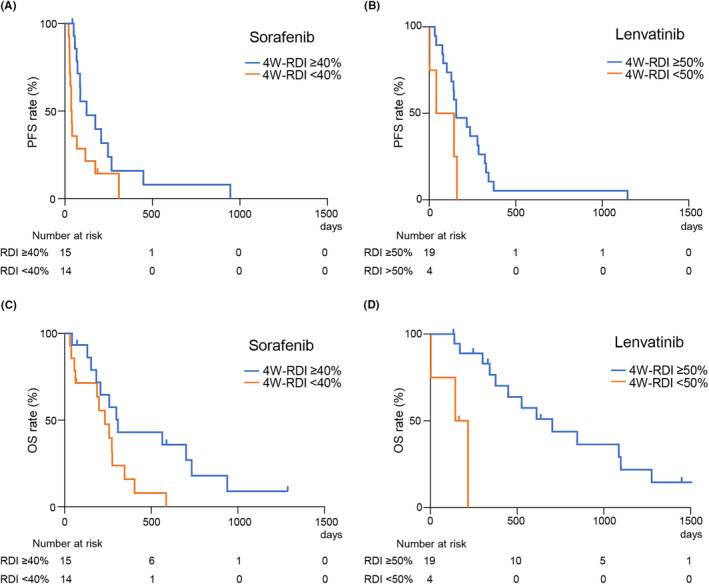
Progression‐free survival (PFS) and overall survival (OS) compared with the high relative dose intensity 4 weeks after treatment (4W‐RDI) and the low 4W‐RDI in elderly patients. (A) The median PFS for sorafenib treatment was 124 and 37.5 days in the 4W‐RDI ≥40% and 4W‐RDI <40% groups, respectively (p < 0.05). (B) The median PFS for lenvatinib treatment was 156 and 92 days in the 4W‐RDI ≥50% and 4W‐RDI <50% groups, respectively (not significant). (C) The median OS for sorafenib treatment was 308 and 233 days in the 4W‐RDI ≥40% and 4W‐RDI <40% groups, respectively (*p* < 0.05). (D) The median OS for lenvatinib treatment was 703 and 180.5 days in the 4W‐RDI ≥50% and 4W‐RDI <50% groups, respectively (*p* < 0.01).

Univariate analysis in factors related to prognosis showed that 4W‐RDI was not associated with longer PFS in non‐elderly patients with unresectable HCC (Table S2). However, univariate and multivariate analyses indicated that high 4W‐RDI was significantly associated with longer PFS in elderly patients (Table S3). Tables 5 shows the results of univariate and multivariate analyses of factors related to OS in the non‐elderly group. The factors significantly associated with longer OS in non‐elderly patients treated with HCC were tumor size <30 mm and the presence of post‐treatment with other MTAs in multivariate analysis. On the contrary, Table 6 shows the results of the univariate and multivariate analyses of factors related to OS in the elderly group. In multivariate analysis, mALBI grade 1/2a, number of the tumors ≤3 and high 4W‐RDI were significantly related to OS in the elderly patients with HCC. Notably, 4W‐RDI ≥40% for sorafenib and 4W‐RDI ≥50% for lenvatinib appears to be a useful prognostic factor for elderly patients with advanced HCC. Furthermore, Figure 3 shows survival curves comparing the prognosis of elderly HCC patients with high and low 4W‐RDI. In elderly patients treated with sorafenib, the high RDI group had significantly prolonged PFS and OS compared to the low RDI group (Figures 3A,C). In elderly patients treated with lenvatinib, PFS was not significantly different in the high 4W‐RDI group compared to the low 4W‐RDI group (Figure 3B). Notably, the high 4W‐RDI group had significantly longer OS on lenvatinib treatment compared to the low 4W‐RDI group (Figure 3D).

**TABLE 5:** Univariate and multivariate analyses of factors associated with overall survival in non‐elderly patients.

	Univariate analysis			Multivariate analysis		
	HR	95% CI	*p*‐Value	HR	96% CI	*p*‐Value
Sex						
Male	1.26	0.63–2.50	N.S.			
BMI						
≥23 kg/m^2^	0.77	0.42–1.42	N.S.			
Past TACE						
No	0.85	0.46–1.60	N.S.			
PS						
0	0.92	0.45–1.87	N.S.			
Etiology						
HCV antibody positive	0.73	0.39–1.35	N.S.			
Treatment started after 2017						
Yes	0.87	0.48–1.59	N.S.			
mALBI grade before treatment						
Grade 1 or 2a	0.59	0.32–1.06	N.S.			
PMI						
≥Cutoff	1.01	0.51–2.00	N.S.			
Maximum size of the tumor						
<30 mm	0.48	0.24–0.94	< 0.05	0.45	0.22–0.90	< 0.05
Number of tumors						
≤3	0.52	0.23–1.18	N.S.			
Major vascular invasion						
No	1.02	0.55–1.92	N.S.			
Extrahepatic metastasis						
No	1.59	0.87–2.93	N.S.			
RDI for the first 4 weeks						
Sorafenib: ≥40%	0.98	0.54–1.78	N.S.			
Lenvatinib: ≥50%						
Post‐treatment with other MTAs						
Yes	0.48	0.26–0.91	< 0.05	1.23	0.66–2.27	N.S.

Abbreviations: BMI, body mass index; CI, confidence interval; HR, hazard ratio; mALBI, modified albumin‐bilirubin; MTAs, molecular target agents; N.S., not significant; PMI, psoas muscle index; RDI, relative dose intensity; TACE, transcatheter arterial chemoembolization.

**TABLE 6:** Univariate and multivariate analyses of factors associated with overall survival in elderly patients.

	Univariate analysis			Multivariate analysis		
	HR	95% CI	*p*‐Value	HR	96% CI	*p*‐Value
Sex						
Male	0.95	0.42–2.19	N.S.			
BMI						
≥23 kg/m^2^	0.80	0.43–1.51	N.S.			
Past TACE						
No	0.79	0.37–1.68	N.S.			
PS						
0	0.86	0.38–1.94	N.S.			
Etiology						
HCV antibody positive	1.22	0.65–2.29	N.S.			
Treatment started after 2017						
Yes	0.82	0.43–1.55	N.S.			
mALBI grade before treatment						
Grade 1 or 2a	0.45	0.23–0.88	< 0.05	0.41	0.20–0.88	<0.05.
PMI						
≥Cutoff	1.41	0.64–3.12	N.S.			
Maximum size of the tumor						
<30 mm	0.51	0.25–1.04	N.S.			
Number of tumors						
≤3	0.36	0.16–0.84	< 0.05	0.35	0.14–0.85	<0.05
Major vascular invasion						
No	1.33	0.55–3.17	N.S.			
Extrahepatic metastasis						
No	1.00	0.51–1.96	N.S.			
RDI for the first 4 weeks						
Sorafenib: ≥40%	0.26	0.12–	< 0.01	0.32	0.14–0.72	< 0.01
Lenvatinib: ≥50%						
Post‐treatment with other MTAs						
Yes	0.3	0.20–0.74	< 0.01	0.62	0.30–1.28	N.S.

Abbreviations: BMI, body mass index; CI, confidence interval; HR, hazard ratio; mALBI, modified albumin‐bilirubin; MTAs, molecular target agents; N.S., not significant; PMI, psoas muscle index; RDI, relative dose intensity; TACE, transcatheter arterial chemoembolization.

## 4 DISCUSSION

Systemic therapy, including MTAs, for patients with advanced HCC has made significant progress, and the increased availability of agents and the widespread use of appropriate management have prolonged the prognosis in HCC patients. Furthermore, the use of MTAs in elderly patients with HCC is expected to increase, but the proportion of elderly patients in clinical trials is small and the evidence is insufficient. In this study, we demonstrate the safety and efficacy of MTAs containing sorafenib and lenvatinib in elderly patients with HCC in a real‐world clinical setting. We also focused on the RDI and reported that maintaining the RDI in the early phase was significantly associated with improved prognosis in elderly patients with HCC; a new finding that is previously unreported.

In this cohort study, no significant difference was observed in the occurrence of any grade AEs with sorafenib or lenvatinib treatment in the elderly compared to non‐elderly HCC patients. Compared to non‐elderly patients, the elderly had significantly lower 4W‐RDI and 8W‐RDI with sorafenib treatment and significantly lower 8W‐RDI with lenvatinib treatment. A higher frequency of anorexia may have influenced the lower RDI for sorafenib and lenvatinib. The occurrence of Grade 3 or higher AEs was not significantly different in elderly patients compared to non‐elderly patients for either sorafenib or lenvatinib treatment, indicating the safety of using MTAs as first‐line treatment in elderly patients with unresectable HCC. The best therapeutic effect for each treatment did not differ between the non‐elderly and elderly groups, but the time to discontinuation of treatment and OS were significantly shorter in the elderly patients treated with sorafenib. For lenvatinib treatment, no significant difference was observed in the rate of conversion to second‐line treatment between the non‐elderly and the elderly groups, nor was there a significant difference in OS. Furthermore, multivariate analysis results revealed that the factor related to longer OS in elderly patients with HCC was high 4W‐RDI in addition to liver reserve and tumor size, with 4W‐RDI ≥40% for sorafenib and 4W‐RDI ≥50% for lenvatinib, which may contribute to good prognosis. Elderly HCC patients should be considered a good indication for systematic therapy when RDI can be maintained in the early phase.

Sorafenib, which was the first MTA to indicate efficacy in unresectable HCC, has been increasingly used in clinical practice since 2008.^24^, ^25^ Sorafenib is the most commonly reported drug in observational studies in elderly patients with unresectable HCC; however, the safety and efficacy of sorafenib are controversial. In a previous study comparing the efficacy of sorafenib in 39 elderly and 151 non‐elderly patients, with 75 years as the borderline age, there was no significant difference in OS and the time to failure in the two groups.^27^ Two studies that defined elderly patients as those aged 70 years or older and compared them to non‐elderly patients also showed that the safety and efficiency of sorafenib treatment in elderly patients with unresectable HCC, with comparable rates of AEs and OS in the two groups.^28^, ^29^ However, some studies have reported contrasting results, with OS for sorafenib treatment of HCC patients significantly worse in the elderly than in the non‐elderly patients. The patient groups included those aged ≥75 years and <75 years in one study, and patients aged ≥70 years and <70 years in another such study.^30^, ^31^ Therefore, the difference in OS of elderly patients with unresectable HCC reported in these studies may be associated with the sorafenib dose and duration of treatment. It has been reported that the dosage of sorafenib should be reduced from the start of treatment in elderly patients with unresectable HCC, particularly those over 80 years of age, as two‐thirds of patients experienced Grade 4 AEs that resulted in a higher discontinuation rate.^31^, ^32^ Furthermore, it has been demonstrated that individual dose adjustment according to AEs prolongs treatment duration and increases the cumulative dose, contributing to longer OS.^33^ Whether the starting dose should be adjusted from the start of sorafenib treatment in the elderly needs to be resolved with further research.

In 2018, lenvatinib was proven non‐inferior to sorafenib for the first time in a study, with OS as the primary endpoint; thus, making lenvatinib the most used drug in clinical practice recently for advanced HCC.^26^ Only 13% of the population evaluated for lenvatinib in the Phase III trial was older than 75 years. A recent retrospective study found that lenvatinib could be used effectively and safely in a cohort of patients with HCC, which consisted of 50 elderly patients aged ≥75 years and 50 non‐elderly patients.^34^ Herein, lenvatinib demonstrated similar PFS, OS, and safety profiles, regardless of patient age. The most frequently occurring AEs in the elderly group were fatigue, anorexia, hypothyroidism, proteinuria, palmoplantar erythematous sensations, and hypertension, all of which were reported to occur as frequently as that in the non‐elderly group. Furthermore, the dose of lenvatinib was significantly lower in the elderly patients, with 28% of the elderly patients and 64% of non‐elderly patients receiving 12 mg of lenvatinib. The starting dose of lenvatinib is 8 or 12 mg depending on body weight, but since no significant difference was found in the BMI between the elderly and non‐elderly groups, a lower dose of lenvatinib may not be related to lower body weight. Further, special dose adjustment other than body weight may be necessary.

Recently, promising therapeutic strategies such as cancer immunosuppressive therapy have improved clinical prognosis for patients with unresectable HCC; and the combination of ICIs and vascular endothelial growth factor inhibitor, Atezo + Bev combination therapy, is now positioned as the first‐line regimen.^35^ In ICI‐based therapy, the combination of tremelimumab plus durvalumab was recently shown to significantly prolong OS compared to sorafenib in the Phase III trials.^36^ Further, several clinical trials of ICI‐based therapies in later‐line treatment are underway, and while some promising results have been shown in nivolumab monotherapy,^37^ and nivolumab plus ipilimumab,^38^ the evidence for clinical use for the elderly patients appears to be insufficient. ICI‐based therapy is promising strategy to enhance the therapeutic effect of MTAs including sorafenib and lenvatinib, or to overcome drug resistance through changes in the tumor immune microenvironment. Therefore, in cases in which optimal RDI cannot be maintained with MTAs, as shown in this study, early conversion to other regimens, especially ICIs, may be a useful therapeutic strategy.

RDI is a useful tool for assessing the tolerability to drug therapy, expressing the total dose of chemotherapy drug per unit time as a percentage of the target dose.^39^ In the patients with metastatic renal cell carcinoma treated with sorafenib refractory to initial therapy, RDI at 1 month is significantly associated with PFS.^40^ In HCC, regorafenib, which has a similar chemical structure to sorafenib, has shown significantly longer PFS and OS in patients with RDI ≥50% at 1 month.^41^ Several studies on lenvatinib treatment of patients with unresectable HCC have also reported that high RDI in the early phase is associated with achieving objective response.^42^, ^43^ Another report suggested that CONUT score was related to clinical outcomes in the patients with HCC treated with lenvatinib, supporting our findings that nutritional status was important for maintaining RDI of MTA treatment.^44^ In addition, a recent study reported that while a high early‐phase RDI (≥80%) of lenvatinib is essential for achieving OR; if maintaining a high early‐phase RDI is difficult, maintaining a moderate RDI (≥40%) to achieve SD should be considered for favorable outcomes in OS and time to progression.^45^ These studies indicate that it is appropriate to adopt RDI ≥40% for sorafenib treatment and RDI ≥50% for lenvatinib treatment as factors associated with prognosis in this study. Furthermore, this study differs from previous studies in that it focused on RDI in the early phase (4 weeks after treatment initiation) as a factor sensitively related to clinical outcomes. We found that maintaining 4W‐RDI was significantly associated with longer OS in elderly patients, but not in non‐elderly patients, and that maintaining RDI at the early phase, especially at 4 weeks, was important in the elderly patients. The decrease in 4W‐RDI in elderly patients with unresectable HCC treated with MTAs was due to early discontinuation or dose reduction, mostly because of AEs occurring in the early phase. Furthermore, in this study, the reasons for MTAs discontinuation in elderly patients with HCC during the entire treatment period were high prevalent, with AEs occurring in 48.3% of patients treated with sorafenib and 47.8% of patients treated with lenvatinib. Therefore, the inability to maintain 4W‐RDI in elderly patients with HCC may reflect the occurrence of AEs and consequently be associated with poor OS.

Combination or sequential therapy with TACE may compensate for the poor tolerability of MTAs in elderly patients with unresectable HCC. Although the clinical efficacy of the combination of MTA and TACE for advanced HCC remains controversial, its potential clinical benefits have been actively reported. In addition, several recent systematic reviews and meta‐analyses have showed that combination therapy with sorafenib and TACE is superior to TACE alone with respect to therapeutic efficacy and safety.^46^, ^47^ The potential efficacy of up‐front lenvatinib and subsequent TACE for intermediate‐stage patients with HCC have been reported.^48^ Furthermore, other studies have indicated that sequential therapy with lenvatinib and TACE has better clinical outcomes than lenvatinib monotherapy.^49^, ^50^ As mentioned above, elderly patients tend to take lower doses of sorafenib and lenvatinib. In combination with TACE, they may be better tolerated in the elderly patients with HCC, and provide a more synergistic therapeutic effect and contribute to improved prognosis.

This study had some limitations. First, this was a retrospective study conducted at a single center. To evaluate the appropriateness of systemic therapy in the elderly patients with HCC, a variety of detailed clinical data needed to be collected, as specific issues to elderly patients, such as dosage, AEs, nutritional status, and skeletal muscle mass, were relevant. Therefore, this study conducted at a single center in our hospital. Second, the number of patients included in this study was small, and a few patients progressed to Atezo + Bev combination therapy, currently the mainstream treatment. Further multicenter studies with many patients who have received various systematic therapeutic drugs, including Atezo+Bev, was needed to verify safety and efficacy for the elderly in clinical settings. Third, failure to maintain 4W‐RDI in elderly patients with HCC may reflect the occurrence of AEs and consequently be associated with poor OS, although further validation with longer follow‐up is needed to clarify this point. However, our findings indicate that the degree of RDI relates to prognosis in systematic therapy drugs, including sorafenib and lenvatinib in elderly patients with HCC.

In conclusion, sorafenib and lenvatinib can be safely administered to elderly patients with advanced HCC. However, their doses tend to be low, and patients with low RDI cannot benefit as much as they should; therefore, alternative treatments such as ICIs or combination with TACE should be considered. Maintaining RDI in the early drug administration phase is vital to assess tolerability and response to MTAs, and contribute to improving the prognosis of elderly patients with advanced HCC.

## AUTHOR CONTRIBUTIONS

**Kyoko Oura:** Conceptualization (equal); data curation (equal); formal analysis (equal); investigation (equal); resources (equal); software (equal); writing – original draft (lead). **Asahiro Morishita:** Conceptualization (supporting); data curation (supporting); formal analysis (supporting); writing – review and editing (supporting). **Kei Takuma:** Investigation (supporting). **Mai Nakahara:** Methodology (supporting). **Tomoko Tadokoro:** Supervision (supporting). **Koji Fujita:** Project administration (supporting). **Shima Mimura:** Validation (supporting); visualization (supporting). **Joji Tani:** Resources (supporting). **Masafumi Ono:** Supervision (supporting). **Takashi Himoto:** Data curation (supporting). **Tsutomu Masaki:** Writing – review and editing (supporting).

## FUNDING INFORMATION

No funding was received.

## CONFLICT OF INTEREST STATEMENT

The authors have no conflict of interest to declare.

## ETHICS STATEMENT

Approval was obtained from the research ethical committee of Kagawa University Faculty of Medicine (Kagawa, Japan) (approval no. 2019‐238).

## Supporting information


Table S1.

## Data Availability

The data that support the findings of the current study are available from the corresponding author upon reasonable request.
