# Supplementary material for: Efficacy and outcome of molecular targeted therapies in elderly patients with hepatocellular carcinoma: Relative dose intensity associated with overall survival
Source: Cancer Med. 2023 Dec 7;12(24):22023–37. doi: 10.1002/cam4.6783 (PMC10757153; doi:10.1002/cam4.6783)
Supplement: Supplementary file 1 — Table S1. [file CAM4-12-22023-s001.docx]

**Table S1. Logistic regression analysis of factors associated with maintaining 4 W-RDI in patients treated with MTAs.**

|  |  | OR | 95% CI | *P*-value |
| --- | --- | --- | --- | --- |
| Sex | Male | 1.83 | 0.74–4.49 | N.S. |
| Age | < 75 years | 0.63 | 0.29–1.35 | N.S. |
| Past TACE | Yes | 1.83 | 0.74–4.49 | N.S. |
| PS | 0 | 0.57 | 0.27–1.23 | N.S. |
| mALBI grade before treatment | Grade 1 | 2.13 | 0.90–5.05 | N.S. |
| CONUT score | 0–4 | 2.73 | 1.21–6.16 | <0.05 |
| PMI | ≥ Cut off | 1.60 | 0.63–4.12 | N.S. |

4W, 4 weeks; RDI, relative dose intensity; TACE, transcatheter arterial chemoembolization; PS. performance status; mALBI, modified albumin-bilirubin; CONUT, controlling nutritional status; PMI, psoas muscle index; OR, odds ratio; CI, confidence interval; N.S., not significant.

**Table S2. Univariate analysis of factors associated with progression-free survival in non-elderly patients.**

|  |  | Univariate analysis | | |
| --- | --- | --- | --- | --- |
|  |  | HR | 95% CI | *P*-value |
| Sex | Male | 0.96 | 0.52–1.78 | N.S. |
| BMI | ≥ 23 kg/m^2^ | 0.87 | 0.50–1.52 | N.S. |
| Past TACE | No | 0.93 | 0.53–1.63 | N.S. |
| PS | 0 | 0.87 | 0.46–1.66 | N.S. |
| Etiology | HCV antibody positive | 0.76 | 0.44–1.38 | N.S. |
| Treatment started after 2017 | Yes | 0.57 | 0.32–1.00 | < 0.05. |
| mALBI grade before treatment | Grade 1 or 2a | 0.58 | 0.34–1.01 | N.S. |
| CONUT score | 0–4 | 1.16 | 0.64–2.12 | N.S. |
| PMI | ≥ Cut off | 1.14 | 0.63–2.07 | N.S. |
| Maximum size of the tumor | < 30 mm | 0.61 | 0.34–1.12 | N.S. |
| Number of tumors | ≤ 3 | 0.67 | 0.34–1.34 | N.S. |
| Major vascular invasion | No | 1.40 | 0.79–2.49 | N.S. |
| Extrahepatic metastasis | No | 1.59 | 0.92–2.75 | N.S. |
| RDI for the first 4 weeks | Sorafenib: ≥ 40%  Lenvatinib: ≥ 50% | 0.75 | 0.44–1.31 | N.S. |
| Post-treatment with other MTAs | Yes | 0.86 | 0.50–1.47 | N.S. |

BMI, body mass index; TACE, transcatheter arterial chemoembolization; mALBI, modified albumin-bilirubin; CONUT, controlling nutritional status; PMI, psoas muscle index; RDI, relative dose intensity; MTAs, molecular target agents; HR, hazard ratio; CI, confidence interval; N.S., not significant.

**Table S3. Univariate and multivariate analyses of factors associated with progression-free survival in elderly patients.**

|  |  | Univariate analysis | | | Multivariate analysis | | |
| --- | --- | --- | --- | --- | --- | --- | --- |
|  |  | HR | 95% CI | *P*-value | HR | 95% CI | *P*-value |
| Sex | Male | 0.38 | 0.17–0.87 | < 0.05 | 0.37 | 0.16–0.84 | < 0.05 |
| BMI | ≥ 23 kg/m^2^ | 0.95 | 0.53–1.70 | N.S. |  |  |  |
| Past TACE | No | 1.03 | 0.51–2.09 | N.S. |  |  |  |
| PS | 0 | 1.44 | 0.68–3.03 | N.S. |  |  |  |
| Etiology | HCV antibody positive | 1.88 | 1.03–3.44 | < 0.05 | 1.46 | 0.78–0.2.74 | N.S. |
| Treatment started after 2017 | Yes | 0.99 | 0.56–1.74 | N.S. |  |  |  |
| mALBI grade before treatment | Grade 1 or 2a | 0.69 | 0.38–1.25 | N.S. |  |  |  |
| CONUT score | 0–4 | 0.86 | 0.48–1.55 | N.S. |  |  |  |
| PMI | ≥ Cut off | 1.45 | 0.71–2.99 | N.S. |  |  |  |
| Maximum size of the tumor | < 30 mm | 0.58 | 0.32–1.06 | N.S. |  |  |  |
| Number of tumors | ≤ 3 | 0.77 | 0.40–1.48 | N.S. |  |  |  |
| Major vascular invasion | No | 1.53 | 0.74–3.19 | N.S. |  |  |  |
| Extrahepatic metastasis | No | 1.45 | 0.75–2.80 | N.S. |  |  |  |
| RDI for the first 4 weeks | Sorafenib: ≥ 40%  Lenvatinib: ≥ 50% | 0.35 | 0.19–0.66 | < 0.01 | 0.35 | 0.18–0.66 | < 0.01 |
| Post-treatment with other MTAs | Yes | 0.61 | 0.34–1.10 | N.S. |  |  |  |

BMI, body mass index; TACE, transcatheter arterial chemoembolization; mALBI, modified albumin-bilirubin; CONUT, controlling nutritional status; PMI, psoas muscle index; RDI, relative dose intensity; MTAs, molecular target agents; HR, hazard ratio; CI, confidence interval; N.S., not significant.
